# Salmonellosis detection and evidence of antibiotic resistance in an urban raccoon population in a highly populated area, Costa Rica

**DOI:** 10.1111/zph.12635

**Published:** 2019-07-29

**Authors:** Mario Baldi, Elías Barquero Calvo, Sabine E. Hutter, Chris Walzer

**Affiliations:** ^1^ Research Institute of Wildlife Ecology University of Veterinary Medicine Vienna Austria; ^2^ Tropical Diseases Research Program, School of Veterinary Medicine National University Heredia Costa Rica; ^3^ Institute of Veterinary Public Health University of Veterinary, Medicine Vienna Austria; ^4^ National Animal Health Service (SENASA) Ministry of Agriculture and Livestock (MAG) Heredia Costa Rica; ^5^ Wildlife Conservation Society Wildlife Health Program Bronx NY USA

**Keywords:** antibiotic resistance, *Procyon lotor*, public health, *Salmonella* sp, zoonosis

## Abstract

Wild animals are involved in zoonotic disease transmission cycles. These are generally complex and poorly understood, especially among animals adapted to life in human ecosystems. Raccoons are reservoirs and effective carriers for infectious agents such as *Salmonella* throughout different environments and contribute to the transference of resistance genes. This study examined the presence of circulating *Salmonella* sp. in a population of raccoons in a tropical urban environment and evaluated resistance to antibiotics commonly used to treat salmonellosis. A total of 97 raccoons of different ages and sex were included in this study. 49% (38–60 CI) of the faecal samples were positive for *Salmonella* spp. The study identified 15 circulating serovars with the most prevalent being *S.* Hartford (7/15), *S.* Typhimurium (4/15) and *S.* Bovismorbificans (4/15). These serovars correspond to the serovars detected in humans with clinical symptoms in Costa Rica. 9.5% of the *Salmonella* strains recovered demonstrated ciprofloxacin resistance, and 7.1% showed resistance to nalidixic acid. This study provides evidence of multiple *Salmonella* serovars circulating in a population of urban raccoons in Costa Rica. Furthermore, the study confirms the existence of antimicrobial resistance to two antibiotics used to treat human salmonellosis. The findings emphasize the role of the raccoon as a reservoir of *Salmonella* in the Greater Metropolitan Area of Costa Rica (GAM) and stress the need for active monitoring of the presence and possible spread in antibiotic resistance due to this peri‐domestic carnivore.

**Impacts**

- Urban raccoons in the Greater Metropolitan Area of Costa Rica interact closely with human beings, enhancing the potential transmission and dissemination of zoonotic pathogens such as *Salmonella*.
- We demonstrate a high prevalence of *Salmonella* strains and a great diversity of *Salmonella* strains in this raccoon population. Additionally, we show evidence of antibiotic resistance to important antibiotics such as ciprofloxacin and nalidixic acid.
- A clearer understanding of the existing risk of close human–raccoon contact will further awareness among health and wildlife management authorities and the general public about the need to avoid close contact with these animals while improving the management of this species in urban areas.

## 1 INTRODUCTION

Wildlife is often the main reservoir of both novel and well‐known zoonotic agents (Rhyan & Spraker, 2010). Nevertheless, the role of wild animals in the sylvatic transmission cycle is sometimes inadequately understood due to a lack of information and the complexity of the systems (Haydon, Cleaveland, Taylor, & Laurenson, 2002; Johnson, 2014; Plowright et al., 2017). Even less is understood about cycles that occur in complex human‐dominated environments such as urban ecosystems (Bradley & Altizer, 2007). Wildlife can adapt and integrate into human settlements and become part of the “natural” wildlife landscape (Luniak, 2008). Data from such ecosystems are fundamental to understanding the potential pathways and dynamics of zoonotic transmission (Adams & Lindsey, 2010; Conover, 2002).

Raccoons (*Procyon lotor*) are one of the species best adapted to colonizing human landscapes (Bateman, Fleming, & Comber, 2012; Zeveloff & Dewitte, 2002). Additionally, they are a natural reservoir for several micro‐ and macroparasites that threaten human health (Bradley & Altizer, 2007; Conover, 2002). *Salmonella* is a well‐reported enterobacterium in raccoon studies in temperate zones (Kumar, 2012). It has previously been pointed out that raccoons play a role in *Salmonella* transmission and dissemination in environments where they are present (Compton et al., 2008; Hoelzer, Moreno Switt, & Wiedmann, 2011; Jardine, Reid‐Smith, Janecko, Allan, & McEwen, 2011).

*Salmonella* is a pathogenic agent that persists in the environment. This is due to the bacteria's specific resistance or more general cross‐resistance mechanisms to a variety of deleterious environmental conditions, but it is also a result of constant environmental contamination by the infected hosts, allowing for effective *Salmonella* expansion into new environments and new vertebrate hosts. This contamination process is associated with the type of vertebrates acting as reservoirs as well as the abiotic conditions, especially in endemic areas (Bondo et al., 2016bb; Hoelzer et al., 2011; Kumar, 2012; Murray, 1991; Spector & Kenyon, 2012).

Furthermore, the presence of antimicrobial resistance (AMR) and the spread of the respective resistance genes represent a serious public health crisis, constraining the effective treatment of affected human patients (O’Neill, 2016). AMR in Costa Rica has a strong economic and public health impact (Ministerio de Salud., 2017), and PAHO has stated that changing to a holistic surveillance of drug resistance helps to fight AMR (Grundmann et al., 2011). Therefore, surveillance should also be extended into wildlife species (Queenan, Häsler, & Rushton, 2016).

The World Health Organization listed Salmonella bacteria as a priority pathogen for promoting research and development (R&D) of new antibiotics (WHO, 2019). Although they are still classified below the high priority group, the increase in quinolone resistance within this group is of serious concern. While wild animals can both harbour and spread resistance genes, they can also serve as sentinels for environmental AMR (Bondo et al., 2016aa; Duncan et al., 2012; Jobbins & Alexander, 2015; Loncaric et al., 2013).

Increasing conflicts due to the presence of raccoons in urban zones of Costa Rica is a recent phenomenon that is attributed to the close proximity of raccoons and human residents inside the most populated areas of Costa Rica (Baldi et al., 2016). The close proximity of humans and raccoons can potentially increase the exchange of pathogens and resistance genes within urban landscapes. This presents a serious public health concern that must be considered and addressed (Bondo et al., 2016aa; Bradley & Altizer, 2007; Conover, 2002). Even more so in Costa Rica, Salmonella epidemiology is limited to reported clinical cases in hospitals (INCIENSA, 2015).

This study determined the prevalence and diversity of endemic *Salmonella* spp strains and provides evidence of antibiotic resistance in urban raccoons within the Great Metropolitan Area (GAM) of Costa Rica.

## 2 MATERIAL AND METHODS

### 2.1 Study area

**Figure 1:**
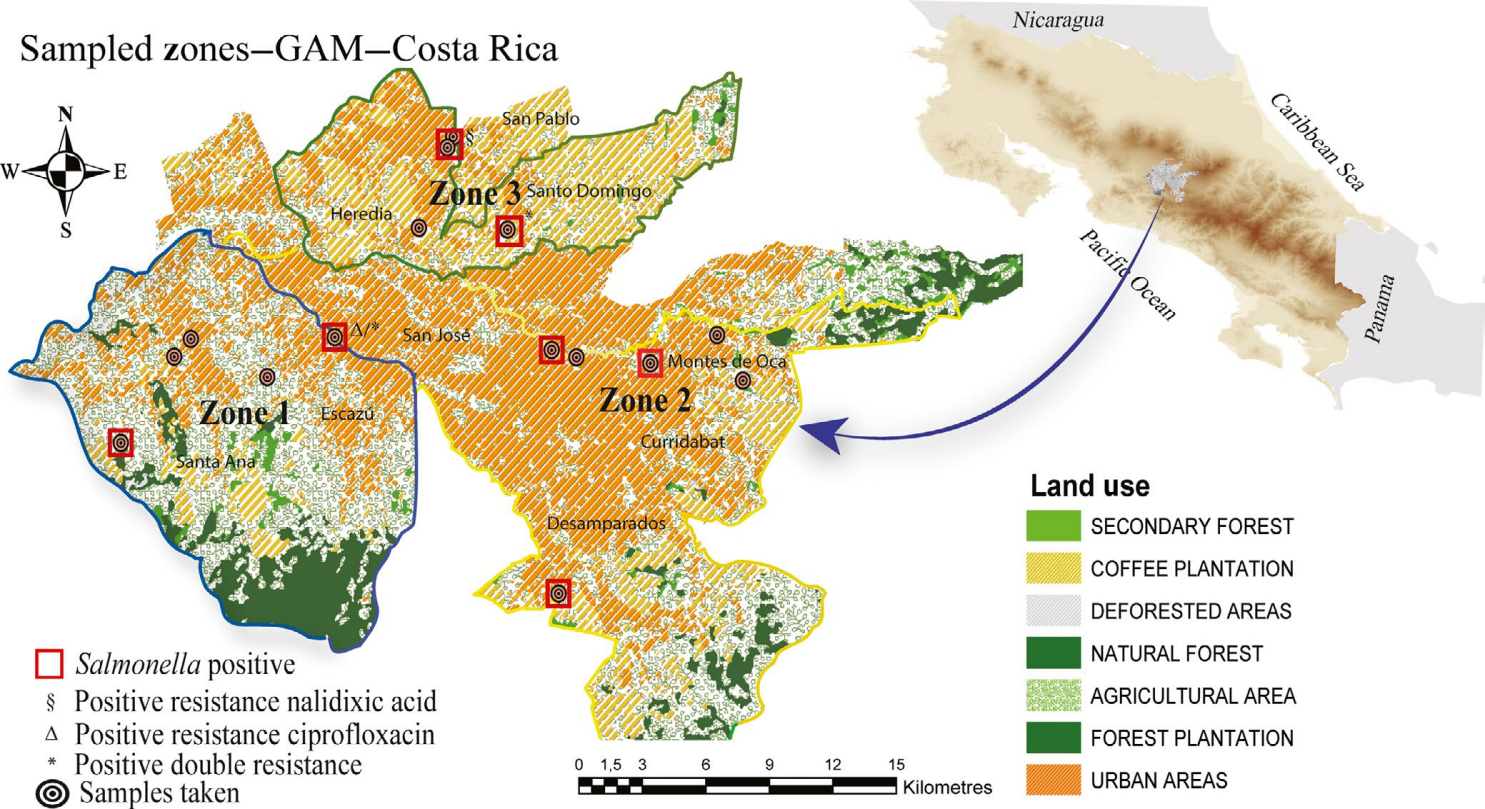
Shows the areas in the Great Metropolitan Area (GAM) where the raccoon samples were taken.

In the period between 2013 and 2016, we captured a total of 97 urban raccoons (*P. lotor*) in 15 different geographic localities within the GAM of Costa Rica (Figure 1). The first 20 individuals were captured opportunistically and used in a pathology‐based study. The remaining 77 animals were sampled, tagged and released. Permission from the Research Ethics Board FCSA‐EMV‐CBA‐007‐2013, Institutional Review Board ACCVC‐OH‐512, Universidad de Costa Rica Institutional Committee for the Use and Care of Laboratory Animals (CICUA‐130‐13) and the Institutional Review Board of MINAE (ACCVC‐OH‐512) was obtained before sampling. Animals were captured by placing traps in small wooded areas near rivers or inside houses and gardens. Traps were baited with bananas, canned cat food or a mixture of both. The traps were set up at night and were visited the following morning. A total of ten Havahart live traps (Lititz, PA, USA) were deployed at each sampling point for a period of three to seven consecutive nights every 2 months for a 12‐month period. Trapping occurred in 15 locations within three zones (Zone 1: Santa Ana‐Escazú, Zone 2: Heredia and Zone 3: San Jose). We captured and removed the first twenty individuals at points where raccoon conflicts had been reported to the local wildlife service authorities. The remaining animals included in the study were part of a mark–capture–recapture programme designed to estimate population density in the GAM.

### 2.2 Biological data collection

Twenty raccoons were euthanized using well‐established standard sedative and anaesthetic protocols. Necropsies were performed at the Laboratory of Pathology at the Universidad Nacional in Costa Rica to determine the presence of zoonotic pathogens (*Salmonella* and other pathogenic agents; Fiette & Slaoui, 2011; Leary & Golab, 2013). Fresh faeces and rectal swabs were collected and immediately processed to determine the presence of *Salmonella* at the Laboratories of Bacteriology at the Universidad Nacional. Assessment of the carcase and internal organs was performed according to previously standardized protocols (Woodford op., 2000) including weight, length and tooth eruption measurements. Tissue samples from internal organs were fixed in 10% neutral buffered formalin for histopathological examination.

The other 77 raccoons were anesthetized using a combination of ketamine–medetomidine 2 and 0.025 mg/kg (Aveco Co. and Domitor® 1 mg/ml; Zoetis) and reversed 45 min later with atipamezole (0.1 mg/kg, Antisedan® 5 mg/ml; Zoetis; West, Heard, & Caulkett, 2014).

Rectal swab samples of all 97 raccoons were collected directly from the rectum and placed in sterile bags (Whirl‐Pak®). All faecal swabs were stored at ~4°C and transported to the previously mentioned laboratories within 3 hr or less. They were then placed in Stuart medium for bacterial culture. The faecal swabs from 11 animals could not be processed due to a problem in labelling. Location, sex, age and weight were recorded for each captured raccoon (Table 1), and individuals were classified as either juvenile or adult based on weight, size and dentition (Grau, Sanderson, & Rogers, 1970).

**Table 1:** Proportion of captured raccoons (*Procyon lotor*) by age, sex and (average) weight

	Males	Females	Total
Adult	40 (5.22%)	42 (5.20%)	82
Juvenile	6 (3.42%)	9 (2.75%)	15
Total	46	51	97

#### 2.2.1 *Salmonella* spp.

A total of 86 rectal swab samples were cultured and characterized via biochemical tests. We followed the methods recommended by the United States Department of Agriculture (USDA) for the isolation and identification of *Salmonella* colonies with some modifications (Laboratory Quality Assurance Staff & USDA/FSIS/OPHS, 2016). Briefly, the primary culture was performed by adding 1 g of stool or a rectal swab to 10 ml of sterile buffered peptone water (Oxoid®) which was then homogenized. Samples were incubated at 35°C ± 2°C for 24 hr, and 1 ml and 0.1 ml aliquots were then transferred to 10 ml of tetrathionate broth (HAJNA; Acumedia®) and 10 ml of Rappaport‐Vassiliadis broth (Acumedia®), respectively. Tubes were incubated in a water bath at 42°C ± 0.5°C for 24 hr. After incubation, an aliquot from each enrichment broth was streaked onto Xylose Lysine Tergitol 4 Agar (XLT4, Acumedia®) and Brilliant Green Sulfa Agar (Acumedia®). Plates were incubated for 24 hr at 35°C ± 2°C. After incubation, *Salmonella* typical colonies were inoculated into Triple Sugar Iron Agar (TSI; Merck®), Lysine Iron Agar (LIA; Oxoid®) and Urea Christensen Agar (Oxoid®) and incubated at 37°C for 24 hr. Compatible biochemical reactions were confirmed with specific *Salmonella* omnivalent O agglutinating antisera (Seiken®) and the VITEK 2 system with the GN identification card following the manufacturer's recommendations (bioMérieux). Finally, typical *Salmonella* colonies were serotyped at the Centro Nacional de Referencia en Bacteriología (CNRB) and Laboratorio Nacional de Servicios Veterinarios (LANASEVE) according to the Kauffmann–White taxonomic scheme using SALMATcor Microagglutination for *Salmonella* flagella serotyping according to Duarte Martínez et al. (2010). Evaluation of the antibiotic susceptibility pattern was performed on each of the *Salmonella* isolates with the VITEK 2 system using the AST‐N279 card (bioMérieux) following the manufacturer's recommendations and using the M100‐S24 sensitivity criteria (‘Clinical Microbiology Procedures Handbook & Fourth Edition’, 2016). The minimum inhibitory concentrations (MICs) for ciprofloxacin and nalidixic acid resistance were determined using E tests (bioMérieux) in Mueller Hinton (BD) agar plates, following the manufacturer's instructions. Breakpoints were determined according to the CLSI (Weinstein, 2019).

### 2.3 Statistical analysis

Descriptive and comparative statistics were performed by using r (R Development Core Team, http://www.r-project.org) to establish the prevalence, and chi‐square test was used to assess the association between *Salmonella* positive animals and their sex, gender and weight.

### 2.4 Geocoding and spatial analysis

A map was generated with the georeferenced points of each sample taken in the three different zones. We assigned a number to the geographic point where the serovar was collected and identified. Serovar information from 15 strains of *Salmonella* was identified and geocoded. The map was generated using arcgis 10.1 software (ERSI).

## 3 RESULTS

A total of 97 raccoons were sampled, but only 86 rectal swab samples could be analysed for *Salmonella* detection during the 20‐month sampling period. Of the total raccoons sampled, 47% (*n* = 46) were males and 54% (*n* = 51) females, 15% were juveniles (15) and 84% (82) adults. None of the raccoons in the study showed any clinical indication of disease, and additionally, gross examination during necropsy (*n* = 20) showed them to be in good health. The body weight had a mean value of 4.87 (CI: 4.50–5.26) kg. Table 1 shows the weight range per sex and age class.

### 3.1 Salmonella

*Salmonella* spp. was cultured from faecal material in 42 of the 86 viable raccoon samples (49.0%, 95% CI: 38–60). Of the 42 positive cultures, we identified a total of 17 serovars, corresponding to the *Salmonella* serovar *S*. Hartford (7/17), followed by *S*. Typhimurium (4/17) and *S*. Bovismorbificans (4/17), *S*. Infantis, *S*. Copenhagen, *S*. San Diego and *S*. Weltevreden (Table 2). Of the 42 positive cultures, in four samples the serovar could not be identified. We found that 88% (15 of 17) of the serovars in raccoons coincided with serovars found in humans, according to the national *Salmonella* surveillance report from 2013 (INCIENSA, 2013). We also found that six raccoon serovars were among the 10 most frequently reported serovars in humans during the same year (INCIENSA, 2013). Four of the serovars (Hartford, Typhimurium, Infantis and Typhimurium var. Copenhagen) were identified in all three sampling areas. The remaining serovars were each found in only one of the sampled areas. The prevalences per sampled zone are shown in Table 3. There was no statistical evidence of associations between sex, gender or weight parameters or with *Salmonella* cases for the animals (*n* = 86) included in these analyses.

**Table 2:** Salmonella serovars detected in the faecal samples of the raccoons

Salmonella serotype	Number of positive samples			
	San Jose	Santa Ana	Heredia	Total
	(*n* = 24)	(*n* = 48)	(*n* = 12)	(*n* = 86)
Hartford	2	5	—	7
Typhimurium^a^^,^ b ^,^ c	3	1	—	4
Bovismorbificans	—	4	—	4
Infantis^c^	2	1	—	3
Typhimurium var Copenhagen^c^	1	2	—	3
Javiana^c^	—	3	—	3
San Diego^c^	2	—	—	2
Jamaica	—	2	—	2
Weltevreden^c^	—	2	—	2
Kentucky^a^	—	1	—	1
Bsilla	—	1	—	1
Shleissheim	—	1	—	1
Paratyphi B	—	1	—	1
Houtenea	1	—	—	1
Minnesota	—	1	—	1
Othmarschen	—	—	1	1
Reading	—	—	1	1
None identified^b^	—	—	4	4
	11 (46%)	25 (52%)	6 (43%)	42

a Ciprofloxacin resistant: 2 isolates.

b Nalidixic acid and ciprofloxacin resistant: 2 isolates.

c Serovar highly detected in humans.

**Table 3:** Salmonella prevalence in raccoon's faeces by location (total of 86 samples)

Zone	San José	Santa Ana	Heredia
Sample animals	*n* = 24	*n* = 48	*n *= 14
Prevalence	0.46	0.52	0.43
CI	0.26–0.67	0.37–0.66	0.19–0.70

Abbreviation: CI, 95% confidence interval.

Antimicrobial resistance was detected in 7.1% (3/42) of the isolates against ciprofloxacin. These three isolates presented a MIC of 0.5 μg/ml. A reduction in the susceptibility against nalidixic acid (MIC: 32 μg/ml) was also detected in one isolate 2.3% (1/42). The rest of the samples were susceptible to all antibiotics tested. Two *Salmonella* isolates obtained from raccoons sampled from two different geographical locations showed resistance to both ciprofloxacin and nalidixic acid. The serovars resistant to ciprofloxacin were identified as *S.* Typhimurium (1), *S.* Kentucky (1) and one *Salmonella* without serovar identification (*S*. none); resistance to nalidixic acid was detected in the following serovars: *S.* Typhimurium (1) and *S.* none (1). Serovars resistant to both antibiotics were as follows: *S.* Typhimurium (1) and *S.* none (1) (Table 2).

## 4 DISCUSSION

Although raccoons are viewed as a natural reservoir for *Salmonella* and *Salmonella* has been previously reported in raccoons in temperate zones, this study establishes, for the first time, the presence of *Salmonella* bacteria and identifies antibiotic resistance to quinolones in raccoon populations in a tropical urban zone in Costa Rica (lat: 9.7489°N).

Previous reports from varying temperate zone urban, rural and natural environments have provided contradictory results as to the prevalence of *Salmonella* serovars in raccoons (Compton et al., 2008; Jardine et al., 2011; Lee et al., 2011; Rainwater et al., 2017; Very et al., 2016). In tropical urban settings, we found *S.* Hartford to have the highest prevalence followed by *S.* Typhimurium and *S.* Bovismorbificans. We did not detect serovar *S.* Newport in our studied animals, even though Compton et al. (2008) described it as the most prevalent serovar in suburban and rural areas Pennsylvania, USA, in contrast to *S.* Typhimurium, which was more prevalent in forest zones. Bondo et al., 2016aa also reported *S.* Newport as the second most prevalent serovar in rural areas of Ontario, Canada, with a very low *S.* Hartford prevalence when compared with the other serovars identified. Concurrently, other authors have reported *S*. Hartford as less prevalent or even absent (Bondo et al., 2016aa; Lee et al., 2011; Very et al., 2016; White, Watson, Hoff, & Bigler, 1975). This contrasts with our findings, where *S*. Hartford was identified at 18.3% (2/11), 20% (5/25) and 0%, respectively, within the three study areas. The reported prevalence *S.* Typhimurium showed that this serovar is among the more consistently recorded serovars in raccoons in rural or natural temperate areas. We found *S.* Typhimurium (16.6%) and *S.* Bovismorbificans (9.5%) to be the second most prevalent serovars in urban areas in Costa Rica. This differs from previous reports in temperate zones (Bondo et al., 2016a, 2016b; Compton et al., 2008; Jardine et al., 2011; Lee et al., 2011; Very et al., 2016; White et al., 1975) and might be associated with the type of urban organization and type of climate, which are very different from the tropical climate and urban organization in Costa Rica.

Our findings show that the serovars *S*. Bovismorbificans and *S*. Typhimurium circulate among the studied raccoon population. *S.* Bovismorbificans is a serovar that has been previously associated with outbreaks in humans, especially in developed countries (Blaylock et al., 2015; Gilsdorf et al., 2005; Hendriksen et al., 2011; Mattila et al., 1998). It can colonize and persist in different species of animals and may contaminate meat products for human consumption (Nógrády, Imre, Kostyák, Tóth, & Nagy, 2010). In Costa Rica, *S.* Bovismorbificans has been found to cause extra‐intestinal and enteric conditions in patients (H. Bolaños, personal communication, July 03 5, 2017). Compton et al. (2008) compared the *Salmonella* pulsed‐field gel electrophoresis (PFGE) results from raccoons and humans and established that some level of overlapping occurs between them. Very et al showed that 16 different PFGE isolates from raccoons were similar to those recovered from clinically ill patients in Pennsylvania, USA. We did not perform a PFGE analysis of our raccoon samples; therefore, it is not possible to establish how many serovars are potentially involved in an exchange or overlap of serovars (Very et al., 2016). Certainly, in our study there is a significant similarity in the number of serovars isolated in raccoons and those reported in humans in the study area (47% national cases; INCIENSA, 2013). Therefore, a future study should compare the PFGE results to explore this relationship.

The presence of different serovars of Salmonella in different areas may reflect a temporary change in serovars in these populations over time. During their study, Jardine et al., 2011 demonstrated that capture–mark–recaptured raccoons in the same locality showed changing serovars and fluctuations in prevalence over time. Our results demonstrated a high diversity of serovars, which could reflect reinfection of the raccoons with new circulating variants in the environment. We did not find a significant sex or age difference among individuals infected with *Salmonella* spp. in our study.

Antimicrobial resistance in raccoon *Salmonella* isolates from our study yielded prevalences similar to other studies (Jardine et al., 2011; Lee et al., 2011; Very et al., 2016). However, only Lee et al., 2011 previously reported resistance to nalidixic acid without resistance to ciprofloxacin in *Salmonella* from raccoons. Nalidixic acid resistance was detected previously in *S.* Infantis (2) and *S.* Typhimurium (1) serovars in raccoons. Both antibiotics are used for salmonellosis and other infections in humans (Pickering, 2012).

While raccoons can be infected with *Salmonella* AMR bacteria from different sources (Bondo et al., 2016a; Martinez, 2009; Radhouani et al., 2014; Rosenblatt‐Farrell, 2009), it appears likely that, in the urban environment, sources are human food, waste and domestic animals (pets) (Baldi et al., 2016). Molina et al., 2016 showed high levels of contamination in animal food and by‐products dedicated to the manufacture of these foods in Costa Rica with different *Salmonella* strains as well as antibiotic resistance genes (Molina et al., 2016). Direct observation of raccoons feeding from rubbish containers used for the disposal of human food plus evidence from stable isotope analysis shows that raccoons in urban areas of Costa Rica regularly feed on human waste food (Sturm & Baldi et al 2017 [untitled work] unpublished raw data). Additionally, raccoons and pets (cats and dogs) share space with a high frequency, as observed in a camera trap study performed in the same study areas (Baldi 2017 [Costa Rican urban raccoon's population estimation by Camera Trap]. unpublished raw data).

In 2013, the Costa Rican AMR surveillance system at CNRB recorded no evidence of resistance against ciprofloxacin in 200 human clinical Salmonella cases. Nevertheless, nalidixic acid resistance was reported in 6% of samples from humans suffering from *S*. Enteritidis, *S*. San Diego and *S*. Kentucky in the same year. This is surprising, as quinolones share some AMR mechanisms (Aldred, Kerns, & Osheroff, 2014). Our AMR study shows that some of the Salmonella isolates have indeed resistance to one or both antibiotics in the same animal. Interestingly, only the *S*. Kentucky isolate was resistant to ciprofloxacin but not nalidixic acid, as was the case in previously observed human cases in Costa Rica. Remarkably, although *S*. Typhimurium is reported to demonstrate ciprofloxacin and nalidixic acid resistance in human studies by different authors (Oteo, Aracil, Alós, & Gómez‐Garcés, 2000; Preethi & Ramanathan, 2016; Roumagnac et al., 2006; Wain et al., 1997), Costa Rica´s national antibiotic resistance surveillance has not found *S*. Typhimurium with such AMR, as noted above. In our study, one isolate was resistant to ciprofloxacin (*S*. Kentucky) and two were resistant to nalidixic acid (none‐identified strain), and two isolates presented resistance to both antibiotics (*S*. Typhimurium/none‐identified strain). Since the mechanisms of resistance are closely related (DNA gyrase [gyrA and gyrB] and topoisomerase IV [parC and pare]; Preethi & Ramanathan, 2016), our results are not surprising.

Nevertheless, plasmid‐mediated quinolone resistance in Salmonella is an alternative resistance mechanism that employs an enzyme to shield DNA gyrase and type IV topoisomerase from quinolone inhibition (Al‐Gallas et al., 2013; van Hoek et al., 2011). The horizontal transmission of qnr genes increases resistance to quinolones such as ciprofloxacin, which could explain why one serovar was only resistant to ciprofloxacin but not to nalidixic acid (Aldred et al., 2014; Ata, Yibar, Arslan, Mustak, & Gunaydin, 2014; Cavaco & Aarestrup, 2009; Ferrari et al., 2013; Pribul et al., 2016).

Most importantly, this shows that a level of AMR is already present in some of the *Salmonella* strains that are circulating in the raccoon population in Costa Rica. This poses the risk of AMR gene transfer as a mechanism of resistance dispersal by different means (physical and biological forces) (Allen et al., 2011; Okeke & Edelman, 2001). Our findings suggest that raccoons could play a role in the dissemination of antibiotic resistance genes in urban areas in Costa Rica.

## 5 CONCLUSION

Our study provides evidence of the presence of various *Salmonella* serovars and related antibiotic resistance in urban raccoons in Costa Rica and highlights the need to monitor raccoons as potential sentinel candidates for *Salmonella* surveillance and environmental antibiotic resistance (Martinez, 2009; Rosenblatt‐Farrell, 2009).

## CONFLICT OF INTEREST

The authors have no conflict of interests.

## References

[zph12635-bib-0001] Adams, C. E. , & Lindsey, K. J. (2010). Urban wildlife management (2nd ed.). London, UK: CRC; Taylor & Francis [distributor]: Boca Raton, FL.

[zph12635-bib-0061] Aldred, K. J. , Kerns, R. J. , & Osheroff, N. (2014). Mechanisms of quinolone action and resistance. Biochemestry, 53(10), 1565–1574. 10.1021/bi5000564 PMC3985860 24576155

[zph12635-bib-0002] Al‐Gallas, N. , Abbassi, M. S. , Gharbi, B. , Manai, M. , Ben Fayala, M. N. , Bichihi, R. , … Ben Aissa, R. (2013). Occurrence of plasmid‐mediated quinolone resistance determinants and rmtB gene in Salmonella enterica serovar enteritidis and Typhimurium isolated from food‐animal products in Tunisia. Foodborne Pathogens and Disease, 10(9), 813–819. 10.1089/fpd.2012.1466 23767853

[zph12635-bib-0003] Allen, S. E. , Boerlin, P. , Janecko, N. , Lumsden, J. S. , Barker, I. K. , Pearl, D. L. , … Jardine, C. (2011). Antimicrobial resistance in generic Escherichia coli isolates from wild small mammals living in swine farm, residential, landfill, and natural environments in southern Ontario, Canada. Applied and Environmental Microbiology, 77(3), 882–888. 10.1128/AEM.01111-10 21131524 PMC3028733

[zph12635-bib-0004] American Society of Microbiology (2016). Clinical microbiology procedures handbook (4th ed.). Washington, DC: ASM Press.

[zph12635-bib-0062] Ata, Z. , Yibar, A. , Arslan, E. , Mustak, K. , & Gunaydin, E. (2014). Plasmid-mediated quinolone resistance in Salmonella serotypes isolated from chicken carcasses in Turkey. Acta Veterinaria Brno, 83(4), 281–286. 10.2754/avb201483040281

[zph12635-bib-0005] Baldi, M. , Alvarado, G. , Smith, S. , Santoro, M. , Bolaños, N. , Jiménez, C. , … Walzer, C. (2016). Baylisascaris procyonis parasites in raccoons, Costa Rica, 2014. Emerging Infectious Diseases, 22(8), 1502–1503. 10.2754/avb201483040281 27433741 PMC4982188

[zph12635-bib-0006] Bateman, P. W. , Fleming, P. A. , & Le Comber, S. (2012). Big city life: Carnivores in urban environments. Journal of Zoology, 287(1), 1–23. 10.1111/j.1469-7998.2011.00887.x

[zph12635-bib-0007] Blaylock, M. , Blackwell, R. , Merid, S. , Jackson, S. , Kotewicz, M. , Gopinath, G. , … Jean‐Gilles Beaubrun, J. (2015). Comparison of Salmonella enterica serovar Bovismorbificans 2011 hummus outbreak strains with non‐outbreak strains. Food Microbiology, 46, 627–634. 10.1016/j.fm.2014.02.016 25475337

[zph12635-bib-0008] Bondo, K. J. , Pearl, D. L. , Janecko, N. , Boerlin, P. , Reid‐Smith, R. J. , Parmley, J. , & Jardine, C. M. (2016a). Epidemiology of Salmonella on the paws and in the faeces of free‐ranging raccoons (*Procyon lotor*) in Southern Ontario, Canada. Zoonoses and Public Health, 63(4), 303–310. 10.1111/zph.12232 26404182

[zph12635-bib-0009] Bondo, K. J. , Pearl, D. L. , Janecko, N. , Boerlin, P. , Reid‐Smith, R. J. , Parmley, J. , & Jardine, C. M. (2016b). Impact of season, demographic and environmental factors on Salmonella occurrence in raccoons (*Procyon lotor*) from swine farms and conservation areas in Southern Ontario. PLoS ONE, 11(9), e0161497 10.1371/journal.pone.0161497 27611198 PMC5017689

[zph12635-bib-0010] Bradley, C. A. , & Altizer, S. (2007). Urbanization and the ecology of wildlife diseases. Trends in Ecology & Evolution, 22(2), 95–102. 10.1016/j.tree.2006.11.001 17113678 PMC7114918

[zph12635-bib-0063] Cavaco, L. M. , & Aarestrup, F. M. (2009). Evaluation of quinolones for use in detection of determinants of acquired quinolone resistance, including the new transmissible resistance mechanisms qnrA, qnrB, qnrS, and aac(6')Ib-cr, in Escherichia coli and Salmonella enterica and determinations of wild-type distributions. Journal of Clinical Microbiology, 47(9), 2751–2758. 10.1128/JCM.00456-09 19571019 PMC2738116

[zph12635-bib-0011] Compton, J. A. , Baney, J. A. , Donaldson, S. C. , Houser, B. A. , San Julian, G. J. , Yahner, R. H. , … Jayarao, B. M. (2008). Salmonella infections in the common raccoon (*Procyon lotor*) in western Pennsylvania. Journal of Clinical Microbiology, 46(9), 3084–3086. 10.1128/JCM.00685-08 18596136 PMC2546707

[zph12635-bib-0012] Conover, M. R. (2002). Resolving human‐wildlife conflicts: The science of wildlife damage management / Michael Conover. Boca Raton, FL: Lewis Publishers.

[zph12635-bib-0013] Duarte Martínez, F. , Sánchez‐Salazar, L. M. , Acuña‐Calvo, M. T. , Bolaños‐Acuña, H. M. , Dittel‐Dittel, I. , & Campos‐Chacón, E. (2010). SALMATcor: Microagglutination for Salmonella flagella serotyping. Foodborne Pathogens and Disease, 7(8), 907–911. 10.1089/fpd.2009.0492 20367066

[zph12635-bib-0014] Duncan, C. , Krafsur, G. , Podell, B. , Baeten, L. A. , LeVan, I. , Charles, B. , & Ehrhart, E. J. (2012). Leptospirosis and tularaemia in raccoons (*Procyon lotor*) of Larimer County, corrected Colorado. Zoonoses and Public Health, 59(1), 29–34. 10.1111/j.1863-2378.2011.01412.x 21824365

[zph12635-bib-0064] Ferrari, R. , Galiana, A. , Cremades, R. , Rodríguez, J. C. , Magnani, M. , Tognim, M. C. B. , … Gloria, R. (2013). Plasmid-mediated quinolone resistance (PMQR) and mutations in the topoisomerase genes of Salmonella enterica strains from Brazil. Brazilian Journal of Microbiology, 44(2), 651–656. 10.1590/S1517-83822013000200046 24294265 PMC3833171

[zph12635-bib-0015] Fiette, L. , & Slaoui, M. (2011). Necropsy and Sampling procedures in rodents In Gautier J.‐C. (Ed.), Drug safety evaluation: Methods and protocols (pp. 39–67). New York, NY: Humana Press. 10.1007/978-1-60761-849-2_3 20972746

[zph12635-bib-0016] Gilsdorf, A. , Jansen, A. , Alpers, K. , Dieckmann, H. , van Treeck, U. , Hauri, A. M. , … Ammon, A. (2005). A nationwide outbreak of Salmonella Bovismorbificans PT24, Germany, December 2004‐March 2005. Euro Surveillance, 10(12), E050324.1. 10.2807/esw.10.12.02667-en 16702643

[zph12635-bib-0017] Grau, G. A. , Sanderson, G. C. , & Rogers, J. P. (1970). Age determination of raccoons. The Journal of Wildlife Management, 34(2), 364 10.2307/3799023

[zph12635-bib-0018] Grundmann, H. , Klugman, K. P. , Walsh, T. , Ramon‐Pardo, P. , Sigauque, B. , Khan, W. , … Stelling, J. (2011). A framework for global surveillance of antibiotic resistance. Drug Resistance Updates, 14(2), 79–87. 10.1016/j.drup.2011.02.007 21482177

[zph12635-bib-0019] Haydon, D. T. , Cleaveland, S. , Taylor, L. H. , & Laurenson, M. K. (2002). Identifying reservoirs of infection: A conceptual and practical challenge. Emerging Infectious Diseases, 8(12), 1468–1473. 10.3201/eid0812.010317 12498665 PMC2738515

[zph12635-bib-0020] Hendriksen, R. S. , Vieira, A. R. , Karlsmose, S. , Lo Fo Wong, D. M. A. , Jensen, A. B. , Wegener, H. C. , & Aarestrup, F. M. (2011). Global monitoring of Salmonella serovar distribution from the World Health Organization Global Foodborne Infections Network Country Data Bank: Results of quality assured laboratories from 2001 to 2007. Foodborne Pathogens and Disease, 8(8), 887–900. 10.1089/fpd.2010.0787 21492021

[zph12635-bib-0021] Hoelzer, K. , Moreno Switt, A. , & Wiedmann, M. (2011). Animal contact as a source of human non‐typhoidal salmonellosis. Veterinary Research, 42(1), 34 10.1186/1297-9716-42-34 21324103 PMC3052180

[zph12635-bib-0022] INCIENSA (2013). ‘Informes Epidemiológicos’. Epidemiological data from National Health Service (Costa Rica) del Instituto Costarricense de Investigación y Enseñanza en Nutricion y Salud. [Online]. (Costa Rica). Retrieved from https://www.inciensa.sa.cr/actualidad/Informes%2520de%2520vigilancia.aspx

[zph12635-bib-0023] INCIENSA (2015). Evaluación Anual Plan Operativo Institucional, 2014. [Online]. Retrieved from https://www.inciensa.sa.cr/inciensa/Inciensa%2520Transparente/Seguimiento%2520Planes%2520Operativos/SEGUIMIENTO%2520ANUAL%2520PLAN%2520OPERATIVO%2520INSTITUCIONAL%2520%25202014.pdf

[zph12635-bib-0024] Jardine, C. , Reid‐Smith, R. J. , Janecko, N. , Allan, M. , & McEwen, S. A. (2011). Salmonella in raccoons (*Procyon lotor*) in southern Ontario, Canada. Journal of Wildlife Diseases, 47(2), 344–351. 10.7589/0090-3558-47.2.344 21441187

[zph12635-bib-0025] Jobbins, S. E. , & Alexander, K. A. (2015). From whence they came–Antibiotic‐resistant *Escherichia coli* in African wildlife. Journal of Wildlife Diseases, 51(4), 811–820. 10.7589/2014-11-257 26221860

[zph12635-bib-0026] Johnson, N. (Ed.) (2014). The role of animals in emerging viral diseases. Amsterdam, the Netherlands and Boston, MA: /Elsevier/AP.

[zph12635-bib-0027] Kumar, Y. (2012). Salmonella ‐ A diversified superbug. Rijeka, Croatia: InTech.

[zph12635-bib-0028] Laboratory Quality Assurance Staff, USDA/FSIS/OPHS (2016). Microbiology laboratory guidebook [Online]. Retrieved from https://www.fsis.usda.gov/wps/portal/fsis/topics/science/laboratories-and-procedures/guidebooks-and-methods/microbiology-laboratory-guidebook/microbiology-laboratory-guidebook

[zph12635-bib-0029] Leary, S. , & Golab, G. C. (2013). AVMA guidelines for the euthanasia of animals: 2013 edition (2013rd ed.). Schaumburg, IL: American Veterinary Medical Association.

[zph12635-bib-0030] Lee, K. , Iwata, T. , Nakadai, A. , Kato, T. , Hayama, S. , Taniguchi, T. , & Hayashidani, H. (2011). Prevalence of Salmonella, Yersinia and Campylobacter spp. in feral raccoons (*Procyon lotor*) and masked palm civets (*Paguma larvata*) in Japan. Zoonoses and Public Health, 58(6), 424–431. 10.1111/j.1863-2378.2010.01384.x 21824337 PMC7165867

[zph12635-bib-0031] Loncaric, I. , Stalder, G. L. , Mehinagic, K. , Rosengarten, R. , Hoelzl, F. , Knauer, F. , & Walzer, C. (2013). Comparison of ESBL–and AmpC producing Enterobacteriaceae and methicillin‐resistant *Staphylococcus aureus* (MRSA) isolated from migratory and resident population of rooks (*Corvus frugilegus*) in Austria. PLoS ONE, 8(12), e84048 10.1371/journal.pone.0084048 24391878 PMC3877145

[zph12635-bib-0032] Luniak, M. (2008). Synurbization ‐ Adaptation of animal wildlife to urban development In Shaw W. W., Harris L. K. & Vandruff L. (Eds.), Proceedings of the 4th International Symposium on Urban Wildlife (pp. 50–55). 2004; Tucson, AZ: University of Arizona.

[zph12635-bib-0033] Martinez, J. L. (2009). Environmental pollution by antibiotics and by antibiotic resistance determinants. Environmental Pollution, 157(11), 2893–2902. 10.1016/j.envpol.2009.05.051 19560847

[zph12635-bib-0034] Mattila, L. , Leirisalo‐Repo, M. , Pelkonen, P. , Koskimies, S. , Granfors, K. , & Siitonen, A. (1998). Reactive arthritis following an outbreak of Salmonella bovismorbificans infection. Journal of Infection, 36(3), 289–295. 10.1016/S0163-4453(98)94243-8 9661939

[zph12635-bib-0035] Ministerio de Salud (2017). Plan de accion nacional de lucha contra la resistencia a los antimicrobianos Costa Rica 2018–2025 [Online]. Retrieved from https://www.ministeriodesalud.go.cr/index.php/vigilancia-de-la-salud/normas-protocolos-y-guias/resistencia-microbiana/3811-plan-de-accion-nacional-de-lucha-contra-la-resistencia-a-los-antimicrobianos-costa-rica-2018-2025/file

[zph12635-bib-0036] Molina, A. , Granados‐Chinchilla, F. , Jiménez, M. , Acuña‐Calvo, M. T. , Alfaro, M. , & Chavarría, G. (2016). Vigilance for Salmonella in feedstuffs available in Costa Rica: Prevalence, serotyping and tetracycline resistance of isolates obtained from 2009 to 2014. Foodborne Pathogens and Disease, 13(3), 119–127. 10.1089/fpd.2015.2050 26682678

[zph12635-bib-0037] Murray, C. J. (1991). Salmonellae in the environment. Revue Scientifique et Technique (International Office of Epizootics), 10(3), 765–785. 1782428

[zph12635-bib-0038] Nógrády, N. , Imre, A. , Kostyák, A. , Tóth, A. , & Nagy, B. (2010). Molecular and pathogenic characterization of *Salmonella enterica* serovar Bovismorbificans strains of animal, environmental, food, and human origin in Hungary. Foodborne Pathogens and Disease, 7(5), 507–513. 10.1089/fpd.2009.0420 20001326

[zph12635-bib-0039] O’Neill, J. (2016). The review on antimicrobial resistance: Tackling drug‐resistant infections globally: Final report and recommendations. [Online]. Retrieved from https://amr-review.org

[zph12635-bib-0040] Okeke, I. N. , & Edelman, R. (2001). Dissemination of antibiotic‐resistant bacteria across geographic borders. Clinical Infectious Diseases, 33(3), 364–369. 10.1086/321877 11438903

[zph12635-bib-0041] Oteo, J. , Aracil, B. , Alós, J. I. , & Gómez‐Garcés, J. L. (2000). High rate of resistance to nalidixic acid in *Salmonella enterica*: Its role as a marker of resistance to fluoroquinolones. Clinical Microbiology and Infection, 6(5), 273–276. 10.1046/j.1469-0691.2000.00058-3.x 11168127

[zph12635-bib-0042] Pickering, L. K. (2012). Red book: 2012 report of the Committee on Infectious Diseases (29th ed.). Elk Grove Village, IL: American Academy of Pediatrics.

[zph12635-bib-0043] Plowright, R. K. , Parrish, C. R. , McCallum, H. , Hudson, P. J. , Ko, A. I. , Graham, A. L. , & Lloyd‐Smith, J. O. (2017). Pathways to zoonotic spillover. Nature Reviews Microbiology, 15(8), 502–510. 10.1038/nrmicro.2017.45 28555073 PMC5791534

[zph12635-bib-0044] Preethi, B. , & Ramanathan, K. (2016). Molecular level understanding of resistance to nalidixic acid in *Salmonella enteric* serovar typhimurium associates with the S83F sequence type. European Biophysics Journal, 45(1), 35–44. 10.1007/s00249-015-1073-2 26329667

[zph12635-bib-0065] Pribul, B. R. , Festivo, M. L. , de Souza, M. M. , & Rodrigues, D. P. (2016). Characterization of quinolone resistance in Salmonella spp. isolates from food products and human samples in Brazil. Brazilian Journal of Microbiology, 47(1), 196–201. 10.1016/j.bjm.2015.04.001 26887245 PMC4822777

[zph12635-bib-0045] Queenan, K. , Häsler, B. , & Rushton, J. (2016). A one health approach to antimicrobial resistance surveillance: Is there a business case for it? International Journal of Antimicrobial Agents, 48(4), 422–427. 10.1016/j.ijantimicag.2016.06.014 27496533

[zph12635-bib-0046] Radhouani, H. , Silva, N. , Poeta, P. , Torres, C. , Correia, S. , & Igrejas, G. (2014). Potential impact of antimicrobial resistance in wildlife, environment and human health. Frontiers in Microbiology, 5, 10.3389/fmicb.2014.00023 PMC3913889 24550896

[zph12635-bib-0047] Rainwater, K. L. , Marchese, K. , Slavinski, S. , Humberg, L. A. , Dubovi, E. J. , Jarvis, J. A. , … Calle, P. P. (2017). Health survey of free‐ranging raccoons (*Procyon lotor*) in Central Park, New York, New York, USA: Implications for human and domestic animal health. Journal of Wildlife Diseases, 53(2), 272–284. 10.7589/2016-05-096 28135131

[zph12635-bib-0048] Rhyan, J. C. , & Spraker, T. R. (2010). Emergence of diseases from wildlife reservoirs. Veterinary Pathology, 47(1), 34–39. 10.1177/0300985809354466 20080482

[zph12635-bib-0049] Rosenblatt‐Farrell, N. (2009). The landscape of antibiotic resistance. Environmental Health Perspectives, 117(6), a244–a250. 10.1289/ehp.117-a244 19590668 PMC2702430

[zph12635-bib-0050] Roumagnac, P. , Weill, F.‐X. , Dolecek, C. , Baker, S. , Brisse, S. , Chinh, N. T. , … Achtman, M. (2006). Evolutionary history of Salmonella typhi. Science, 314(5803), 1301–1304. 10.1126/science.1134933 17124322 PMC2652035

[zph12635-bib-0051] Spector, M. P. , & Kenyon, W. J. (2012). Resistance and survival strategies of Salmonella enterica to environmental stresses. Food Research International, 45(2), 455–481. 10.1016/j.foodres.2011.06.056

[zph12635-bib-0052] van Hoek, A. H. A. M. , Mevius, D. , Guerra, B. , Mullany, P. , Roberts, A. P. , & Aarts, H. J. M. (2011). Acquired antibiotic resistance genes: An overview. Frontiers in Microbiology, 2, 203 10.3389/fmicb.2011.00203 22046172 PMC3202223

[zph12635-bib-0053] Very, K. J. , Kirchner, M. K. , Shariat, N. , Cottrell, W. , Sandt, C. H. , Dudley, E. G. , … Jayarao, B. M. (2016). Prevalence and spatial distribution of Salmonella infections in the Pennsylvania raccoon (*Procyon lotor*). Zoonoses and Public Health, 63(3), 223–233. 10.1111/zph.12222 26272724

[zph12635-bib-0054] Wain, J. , Hoa, N. T. T. , Chinh, N. T. , Vinh, H. , Everett, M. J. , Diep, T. S. , … Parry, C. M. (1997). Quinolone‐resistant Salmonella typhi in Viet Nam: Molecular basis of resistance and clinical response to treatment. Clinical Infectious Diseases, 25(6), 1404–1410. 10.1086/516128 9431387

[zph12635-bib-0055] Weinstein, M. P. (Ed.) (2019). Performance standards for antimicrobial susceptibility testing (29th ed.). Wayne, Pennsylvania: Clinical and Laboratory Standards Institute.

[zph12635-bib-0056] West, G. , Heard, D. , & Caulkett, N. (2014). Zoo animal and wildlife immobilization and anesthesia. Ames, IA: John Wiley & Sons Inc.

[zph12635-bib-0057] White, F. H. , Watson, J. J. , Hoff, G. L. , & Bigler, W. J. (1975). *Edwardsiella tarda* infections in Florida raccoons, *Procyon lotor* . Archives of Environmental Health: an International Journal, 30(12), 602–603. 10.1080/00039896.1975.10666788 1200720

[zph12635-bib-0058] WHO (2019). WHO publishes list of bacteria for which new antibiotics are urgently needed [Online]. Retrieved from https://www.who.int/news-room/detail/27-02-2017-who-publishes-list-of-bacteria-for-which-new-antibiotics-are-urgently-needed

[zph12635-bib-0059] Woodford, M. H. (op. 2000). Post‐mortem procedures for wildlife veterinarians and field biologists. Paris, France: Office International des Epizooties, Care for the Wild International, The World Conservation Union.

[zph12635-bib-0060] Zeveloff, S. I. , & Dewitte, E. (2002). Raccoons: A natural history. Vancouver, BC: UBC Press.

